# A targeted approach for evaluating preclinical activity of botanical extracts for support of bone health

**DOI:** 10.1017/jns.2014.5

**Published:** 2014-05-13

**Authors:** Yumei Lin, Mary A. Murray, I. Ross Garrett, Gloria E. Gutierrez, Jeffry S. Nyman, Gregory Mundy, David Fast, Kevin W. Gellenbeck, Amitabh Chandra, Shyam Ramakrishnan

**Affiliations:** 1 Nutrilite Health Institute, 5600 Beach Boulevard, Buena Park, CA 90622, USA; 2 OsteoScreen Ltd, 2040 Babcock Road, San Antonio, TX 78023, USA; 3 9909 Charthouse Cove, Austin, TX 78730, USA; 4 Southwest Research Institute, 6220 Culebra Road, San Antonio, TX 78238, USA; 5 Department of Orthopaedic Surgery and Rehabilitation, Vanderbilt University Medical Center, Nashville, TN 37232, USA; 6 Vanderbilt University Medical Center, Nashville, TN 37232, USA; 7 Access Business Group, 7575 East Fulton Avenue, Ada, MI 49355, USA; 8 The Himalaya Drug Company, Makali, Tumkur Road, Bangalore – 562123, India

**Keywords:** Botanical extracts, Receptor activator of nuclear factor-κB ligand, Bone morphogenetic protein-2, Bone formation, AR, anti-resorptive sample, BF, bone formation sample, BMD, bone mineral density, BMP, bone morphogenetic protein, µCT, micro-computed tomography device, OVX, ovariectomised, PTH, parathyroid hormone, RANKL, receptor activator of nuclear factor-κB ligand, SHAM, sham-operated, vBMD, volumetric bone mineral density

## Abstract

Using a sequential *in vitro*/*in vivo* approach, we tested the ability of botanical extracts to influence biomarkers associated with bone resorption and bone formation. Pomegranate fruit and grape seed extracts were found to exhibit anti-resorptive activity by inhibiting receptor activator of nuclear factor-κB ligand (RANKL) expression in MG-63 cells and to reduce IL-1β-stimulated calvarial ^45^Ca loss. A combination of pomegranate fruit and grape seed extracts were shown to be effective at inhibiting bone loss in ovariectomised rats as demonstrated by standard histomorphometry, biomechanical and bone mineral density measurements. Quercetin and licorice extract exhibited bone formation activity as measured by bone morphogenetic protein-2 (BMP-2) promoter activation, increased expression of BMP-2 mRNA and protein levels, and promotion of bone growth in cultured mouse calvariae. A combination of quercetin and licorice extract demonstrated a potential for increasing bone mineral density in an intact female rat model as compared with controls. The results from this sequential *in vitro*/*in vivo* research model yielded botanical extract formulas that demonstrate significant potential benefits for bone health.

Bone is a dynamic tissue in a constant cycle of resorption and formation. Established bone is resorbed by osteoclasts while osteoblasts build bone in the excavated site. In children, there is rapid bone growth and remodelling; however, in adults, there are only modest changes in bone size but continuous remodelling. In adults, the remodelling process renews approximately 10 % of the bone each year and the entire skeleton is renewed in roughly 7 to 10 years^(^^1^^)^. Bone mass changes with age, peaking in adults aged 20 to 30 years and declining after 50 years of age. The loss of bone mass associated with ageing can result in an increased risk of fracture and osteoporosis^(^^2^^)^.

Osteoporosis, a chronic progressive disorder, has become a global health issue due to increasing life expectancies. Data from the National Health and Nutrition Examination Survey (NHANES) estimates that more than 10 million Americans over the age of 50 years have osteoporosis, including 7·8 million women and 2·3 million men. Another 33·6 million Americans over the age of 50 years have osteopenia, low bone mass, which increases their risk for osteoporosis^(^^3^^)^. While osteoporosis is a diagnosed and often medically treated condition, therapeutic intervention for osteopenia is currently controversial. Without treatment, this condition will ultimately progress to osteoporosis. There is also evidence that individuals with osteopenic bone mineral density (BMD) levels are susceptible to fragility fractures^(^^4^^)^.

While the increase in the incidence of osteoporosis and osteopenia is due in part to increased lifespan, the Western diet may also play a role^(^^5^^,^^6^^)^. It is widely accepted that an adequate intake of dietary Ca, vitamin D and protein are important for maintaining bone health. However, in recent research, a positive correlation between fruit and vegetable intake and BMD has been suggested^(^^7^^,^^8^^)^, and research into the basis for this correlation points to a role for polyphenolic compounds found in plants^(^^2^^,^^9^^)^.

Research on bone physiology and disease has identified biomarkers that provide targets for measuring the effects of drugs or nutritional intervention on bone health. Osteogenic inducing factors include the bone morphogenetic proteins (BMP), members of the transforming growth factor-β family. BMP-2 and BMP-4 have been established as crucial for the progression and maturation of osteogenesis^(^^10^^)^. The production of recombinant human BMP-2 has led to the ability to manufacture large enough quantities for the clinical treatment of fractures and spine fusion^(^^11^^)^.

Receptor activator of nuclear factor-κB ligand (RANKL), a cytokine of the TNF family pathway, is known to play a role in the maturation of osteoclasts, a key step in the induction of bone resorption, and is a predominant target for therapeutic agents developed for the treatment of osteoporosis^(^^10^^)^. The interaction between receptor activator of nuclear factor-κB (RANK) and RANKL leads to the maturation and activation of osteoclasts. Therefore, agents inhibiting RANKL activity would be expected to increase bone density, volume and strength.

Because of the role of regular consumption of fruits and vegetables in bone health, the cost and side-effect concerns associated with common osteoporosis treatments, as well as a philosophy of wellness rather than treating a disease, we designed a structured approach to explore the use of botanical extracts to maintain bone health. Botanical extracts were selected based upon available scientific literature, and were evaluated for bone formation and/or anti-resorptive activity using a series of *in vitro* and *in vivo* assays. The present paper describes the structured approach we took to evaluate select botanical extracts which could lead to the development of anti-resorptive and bone formation nutraceutical formulations.

## Materials and methods

### Botanical extracts and test compounds

The sources and characteristics of the botanical extracts selected for screening are described in Table 1. The quality and standardisation of the extracts were verified using the appropriate analytical methods^(^^12^^)^.

**Table 1.** Botanical extracts screened for effect on biomarkers of bone resorption or formation

Common name	Species	Plant part	Standardisation (minimum specification)
Dong quai	*Angelica sinensis*	Root	Not standardised
Eleuthero	*Eleurococcus senticosus*	Root/rhizome	0·75 % Eleutherosides
Fava d'anta	*Dimorphandra mollis*	Fruit	86 % Quercetin
Ginkgo	*Ginkgo biloba*	Leaf	24 % Flavonol-glycosides, 6 % terpene lactones
Grape	*Vitis vinifera*	Seed	40 % Polyphenolics (spectrophotometery)
Green tea	*Camellia sinensis*	Leaf	40 % Epigallo-catechin gallate
Licorice	*Glycyrrhiza glabra*	Rhizome	0·05 % Glabridin, about 1 % glycyrrhizic acid
Pomegranate	*Punica granatum*	Fruit	40 % Ellagic acid
Rehmannia	*Rehmannia glutinosa*	Root	1 % Catapol
Sophora	*Sophora japonica*	Flower/fruit	Not standardised

Ipriflavone, a synthetic isoflavone, was obtained from TSI Health Sciences; alendronate sodium (Fosamax^®^, 70 mg free acid equivalent in 75 ml oral solution) and simvastatin (Zocor^®^) were purchased from Merck and Co. The parathyroid hormone (PTH) used was rat PTH(1-34) from Bachem.

### Cell culture

Osteoblast-like MG-63 human osteosarcoma cells (ATCC (American Type Culture Collection), Manassas, VA, USA) were cultured in a 37°C, 10 % CO_2_ incubator in phenol red-free Dulbecco's modified Eagle's medium supplemented with 10 % fetal bovine serum, 1 % penicillin/streptomycin, 1 % amphotericin B, and 2 mm-l-glutamine. MG-63 cells at passage 10 or less were used for the experiments. Clonal osteoblast cells (2T3 cells; ATCC) stably transfected with the murine BMP-2 promoter (–2712/+165) linked to firefly luciferase cDNA were cultured in a 37°C, 5 % CO_2_ incubator in α-minimum essential medium (α-MEM) supplemented with 10 % fetal calf serum (FCS), 1 % penicillin/streptomycin, 1 % amphotericin B and 2 mm-l-glutamine. The stably transfected 2T3 cells at passage 10 or less were used for the experiments.

### Receptor activator of nuclear factor κ-B ligand expression assay

Bioassay testing for the development of an anti-resorptive formula was initiated by screening the botanical extracts for their ability to suppress RANKL expression. MG-63 cells were cultured in phenol red-free medium with 0·5 % fetal bovine serum to eliminate any potential oestrogenic effects due to phenol red. The MG-63 cells were incubated overnight with botanical extracts (1, 10 and 100 µg/ml) with the addition of human recombinant IL-1β (10 ng/ml) to induce RANKL expression. The MG-63 cells were assayed for RANKL mRNA using quantitative real-time PCR, using DLUX primers for human RANKL and Superscript III Platinum reagents (Invitrogen). The expression of RANKL was normalised to glyceraldehyde 3-phosphate dehydrogenase (GAPDH), a housekeeping gene.

### Bone morphogenetic protein-2 gene expression assay

The ability of the botanical extracts to activate BMP-2 gene expression was determined using MG-63 cells in phenol-red free medium. The cells were treated with 10 µg/ml sample extracts and incubated overnight. BMP-2 mRNA levels were quantified by real-time PCR using DLUX primers for human BMP-2 and Superscript III Platinum reagents. The results were normalised to expression of the reference gene, GAPDH. Due to the inherent variability of real-time PCR for relative quantification, we considered a greater than two-fold increase in BMP-2 expression to be our threshold of significance.

### Bone morphogenetic protein-2 luciferase promoter assay

A clonal osteoblast cell line (2T3 cells) was stably transfected with the murine BMP-2 promoter (–2712/+165) linked to firefly luciferase cDNA, as previously described^(^^13^^)^. Briefly, 2T3 Luc cells were placed in a ninety-six-well plate at a concentration of 5 × 10^3^ cells per well in α-MEM supplemented with 10 % FCS and incubated for 48 h. The medium was aspirated, the cells washed, and 200 µl of the test sample in α-MEM, supplemented with 2·5 % FCS, were added to each well. The cells were lysed and luciferase activity was measured via a luciferase assay kit (Promega) using a Turner Designs Luminometer Model TD 20/20. Activation of the BMP-2 promoter was considered a positive response when an increase in luminescence above baseline level was observed. The extracts were tested at a range of concentrations (0·05 to 100 µg/ml) and a two-fold relative change in protein promoter was our threshold of significance.

### Bone morphogenetic protein-2 ELISA assay

Bone-forming activity was further characterised by quantifying BMP-2 protein produced by treated MG-63 cells. Cells were treated overnight with botanical extract at 1, 10 and 100 µg/ml. The cell supernatant fractions were then quantified for BMP-2 levels using a commercially available ELISA assay (R&D Systems).

### Calvarial anti-resorption and bone formation assays

Pregnant female Swiss white mice were purchased from Harlan Laboratories and housed in Laboratory Animal Resources at the University of Texas, Health Science Center at San Antonio. Murine calvarial tissues were harvested from the 4-d-old neonatal pups and incubated with botanical extracts as part of the testing strategy for the development of both anti-resorptive and bone formation formulas. These *in vitro* assays were performed by Osteoscreen. All protocols were reviewed and approved by The Institutional Animal Care and Use Committee of The University of Texas Health Science Center at San Antonio for compliance with regulations.

The murine calvarial (skullcap) bone anti-resorptive assay was performed as previously described^(^^14^^,^^15^^)^. Briefly, pregnant female mice were injected intraperitoneally on gestational day 18 with 25 µCi ^45^CaCl_2_, killed 24 h later, and the fetuses removed. The 4-d-old neonatal pups were killed and the calvariae removed and bisected following removal of the loose subcutaneous tissue. The half-calvariae were cultured in 1 ml BGJb medium (Gibco) for 24 h at 37°C, in a 5 % CO_2_ humidified incubator to allow for exchange of loosely complexed ^45^Ca with the stable Ca in the medium. Calvariae were then cultured for 3 d in fresh BGJb medium supplemented with 0·1 % bovine serum albumin containing botanical extracts and IL-1β (10^−10^ m). A total of four to six calvariae were used for each treatment group. Bone resorption was determined by measuring the percentage of total incorporated radioactivity released from the bones during the culture period.

The murine calvarial bone formation assay was performed as previously described^(^^15^^,^^16^^)^. In brief, the calvariae from 4-d-old neonatal pups of Swiss white mice were excised and incubated with select botanical extracts for 7 d. BMP-2 (50 ng/ml) and simvastatin (0·25, 0·5 and 1·0 µm) were used as positive controls. At the end of the incubation period, 4 µm sections of the calvariae were prepared and morphological assessment completed. Digital images of the murine calvariae sections were taken and histomorphometric analysis performed on the images using Image Pro Plus (Media Cybernetics, Inc.). The total and new bone areas (expressed as mm^2^ × 10^−3^) were determined on all images across the calvarial section.

### *In vivo* anti-resorption and bone formation studies

Animal studies were conducted in order to determine the effects of the botanical extracts on bone resorption and formation *in vivo*. Animals were purchased from Harlan Laboratories and housed in Laboratory Animal Resources at the University of Texas, Health Science Center at San Antonio. All protocols were reviewed and approved by The Institutional Animal Care and Use Committee of The University of Texas Health Science Center at San Antonio for compliance with regulations. The study endpoints included histological analysis, biomechanical measurements and BMD.

Female Sprague–Dawley rats (3 months old; 200–250 g) were used for the anti-resorptive screening experiments. All animals were allowed free access to food and water and were fed a standard laboratory chow diet before ovariectomy. Rats were weight-matched and randomly placed into one of nine study groups. All rats were then either bilaterally ovariectomised (OVX) or sham-operated (SHAM). In the SHAM group, the ovaries were bilaterally exposed and handled, but not removed. To verify complete removal of the ovaries, at the end of the experimental period the uterus was excised and weighed.

Following recovery from surgery (approximately 24 h), the animals were fed a diet of either 15 g/d normal rat chow or a custom diet of normal rat chow mixed with select botanical extracts for a period of 35 d. The OVX rats were randomised into eight groups: six different botanical preparations (*n* 15/group), a vehicle control (*n* 14) and a positive control (alendronate, 0·5 mg/kg per d, given by oral administration, *n* 15). SHAM rats (*n* 5) were fed a control diet and acted as controls for comparing loss of bone mass of OVX rats. Animals were killed on day 35 following treatment and the bones processed for analysis.

To assess the effects of the botanical extracts on bone formation, 3-month-old Sprague–Dawley intact virgin female rats (200–250 g) were fed 15 g/d normal chow or a combination of normal chow and chow containing botanical extracts for a period of 35 d. The rats were randomly assigned to one of eight groups (*n* 15): six different botanical preparations, a vehicle control and a positive control (PTH 50 µg/kg administered by intra-muscular injection, three times per week). To quantify bone formation, calcein (10 mg/kg) and tetracycline (50 mg/kg) were administered intramuscularly to all animals at 2 and 7 d before killing, respectively. Animals were killed on treatment day 35 and their bones processed for analysis.

All animal chow (control diet TD 06261 and custom chow containing botanical extracts) was prepared by Harlan Laboratories (Teklad Lab Animal Diets). Compositions of the custom chows containing botanical extracts for the anti-resorption study are given in Table 2, and for the bone formation study in Table 3. The custom and normal rat chows were tested by established analytical methods^(^^12^^)^ to confirm that the active ingredients were positively identified in the samples and were not present in the control diet. The relative concentrations of analytes for the custom diets were confirmed but, due to extremely low levels of analytes, absolute quantification was not conducted.

**Table 2.** Effects of botanical extracts on bone resorption in ovariectomised female Sprague–Dawley rats

	Botanicals† (mg/kg body weight)			Histomorphometry (%)	Biomechanical (%)		DXA (%)	µCT (%)			
Sample	P	GS	I	%BA/TV	S	MF	BMD	vBMD	%BV/TV	Tb.N	Tb.Sp
AR-1	51	5·1	0	↑ 61·4*	↑ 4·9	↓ 0·4	↑ 0·6	↑ 20·6	↑ 107·4*	↑ 58·3*	↓ 47·1*
AR-2	127	12·7	0	↑ 13·5	↑ 0·6	↑ 8·3*	↑ 7·8*	↑ 32·0*	↑131·6*	↑ 99·5*	↓ 52·4*
AR-3	203	20·3	0	↑ 13·1	↑ 16·0*	↑ 8·1*	↑ 10·2*	↑ 32·0*	↑ 144·7*	↑ 121·5*	↓ 63·6*
AR-4	0	0	61	↓ 12·9	↑ 4·6	↓ 0·6	↑ 6·0*	↑ 25·2*	↑ 137·2*	↑ 116·7*	↓ 58·1*
AR-5	51	5·1	61	↑ 6·4	↑ 10·2	↑ 0·9	↑ 6·0*	↑ 22·4	↑ 156·2	↑ 95·7	↓ 42·2*
AR-6	127	12·7	61	↑ 41·7	↑ 3·3	↑ 3·0	↑ 4·8*	↑ 0·7	↑ 11·1	↑ 14·4	↓ 18·2
ALD‡	0	0	0	↑ 214·1*	↑ 0·2	↓ 0·4	↑ 35·3*	↑ 123·5*	↑ 632·2*	↑ 313·4*	↓ 88·0*

DXA, dual-energy X-ray absorptiometry; µCT, micro-computed tomography device; P, pomegranate; GS, grape seed; I, ipriflavone; %BA/TV, percentage bone area/total volume; S, stiffness; MF, maximum force; BMD, bone mineral density; vBMD, volumetric bone mineral density; %BV/TV, percentage bone volume/total volume; Tb.N, trabecular number; Tb.Sp, trabecular separation; AR, anti-resorptive sample; ↑, increase; ↓, decrease; ALD, alendronate.

* Statistical difference compared with vehicle control (*P* < 0·05).

† Rats were fed 15 g/d normal chow or normal chow containing botanical extracts for a period of 35 d.

‡ ALD-treated rats were given 0·5 mg/kg per d by oral administration.

**Table 3.** Effects of botanical extracts on bone formation in 3-month-old intact female Sprague–Dawley rats

	Botanicals† (mg/kg body weight)		Histomorphometry (%)		Biomechanical (%)		µCT (%)			
Sample	Q	L	%BV/TV	BFR	S	MF	vBMD	%BV/TV	Tb.N	Tb.Sp
BF-1	25	25	↑ 3·0	↑ 4·10	↓ 6·2	↓ 8·8	↑ 17·3	↑ 32·0*	↑ 15·1*	↓ 21·9*
BF-2	51	51	↓ 6·4	↑ 15·8	↓ 3·8	↓ 10·1	↑ 71·3*	↑ 33·3*	↑ 13·8*	↓ 22·4*
BF-3	25	13	↑ 47·6*	↑ 51·4*	0	↓ 4·9	↑ 19·9	↑ 15·4	↑ 7·0	↓ 9·2
BF-4	51	25	↑ 11·2	↑ 16·6	↓ 2·4	↓ 4·0	↑ 30·7	↑ 12·6	↑ 4·9	↓ 7·6
BF-5	102	51	↑ 29·0*	↑ 20·3	↓ 6·7	↓ 6·8	↑ 74·5	↑ 24·8*	↑ 14·1*	↓ 19·2*
BF-6	102	20	↓ 6·6*	↑ 3·2	↓ 4·5	↓ 2·8	↑ 19·5	↑ 9·4	↑ 3·8	↓ 4·3
PTH‡	0	0	↑ 55·8*	↑ 45·8*	↑ 1·8	↓ 4·1	↑ 86·0*	↑ 32·2*	↑ 24·8*	↓ 28·3*

µCT, micro-computed tomography device; Q, quercetin; L, licorice; %BV/TV, percentage bone volume/total volume; BFR, bone formation rate; S, stiffness; MF, maximum force; vBMD, volumetric bone mineral density; Tb.N, trabecular number; Tb.Sp, trabecular separation; BF, bone formation sample; PTH, parathyroid hormone.

* Statistical difference compared with vehicle control (*P* < 0·05).

† Rats were fed 15 g/d normal chow or normal chow containing botanical extracts for a period of 35 d.

‡ PTH-treated rats were given 50 µg/kg per d.

To complete histological measurements in the anti-resorptive studies, the tibia and femur bones were removed, cleaned of soft tissue, fixed in 10 % formalin for 48 h, and stored in 70 % ethanol before preparation for histological analysis by embedding in paraffin. The proximal tibia was examined using a semi-automated Osteomeasure System (Osteometrics Inc.) with a digitising pad. Cancellous bone volume was quantified and expressed as bone/tissue area. Bone volume, trabecular number and separation, cell number and dynamic parameters were also determined as previously described^(^^17^^)^.

To perform histological measurements, femurs were fixed in 10 % formalin, and then transferred to 70 % ethanol, dehydrated in increasing concentrations of ethanol and embedded in plastic (methyl methacrylate). Sections (7 µm) were prepared for histomorphometric analysis. Two consecutive sections were prepared for the analysis of each sample, one unstained for visualisation of the fluorochromes, calcein and tetracycline, and the other dyed with Von Kossa stain. Structural and dynamic measurements were processed using a bone histomorphometry system with a digitising tablet (Osteomeasure; Osteometrics Inc.) attached to a Nikon E-400 fluorescent microscope. Trabecular bone volume was assessed in Von Kossa-stained sections.

Bone formation and mineral apposition rates were measured in unstained sections. Measurements were confined to the secondary spongiosa of the distal femur, starting at 1 mm below the growth plate to exclude the primary spongiosa. The rate of bone formation (µm^2^/mm^3^ per d) was calculated from the extent of bone surface labelled with tetracycline and, where present, the distance between the two fluorochromes, calcein and tetracycline. Bone volume was expressed as the percentage of bone volume in the area measured. Mineral apposition rate was the mean interlabel distance divided by the time interval (5 d) between the two fluorochromes administered. The bone surface referent/bone formation rate was expressed as mm^3^/µm^2^ per d.

Biomechanical measurements were taken after femurs were removed and stored frozen. On the day of testing, the femurs were thawed to room temperature and remaining soft tissue removed. The bones were subjected to a three-point bending biomechanical testing using an EnduraTEC mechanical testing system (Elf 3300; Bose Corporation). Each rat femur was placed horizontally on the support rollers and force-displacement recorded as the indenter travelled at a rate of 3 mm/min into the femur midshaft. Biomechanical properties (stiffness and maximum force) were derived directly from the load deformation curves.

To determine BMD, excised femurs were scanned using a Lunar PIXImus dual-energy X-ray absorptiometry densitometer (GE Medical Systems). Results were expressed as g/cm^2^ of bone area of the proximal tibia. Total bone and secondary spongiosa areas were quantified.

The distal femurs were scanned using a micro-computed tomography micro-CT device (µCT; SCANCO Medical) 6 mm from the distal head at 30 µm. Measurements included volumetric BMD (vBMD), percentage bone volume/total volume, and trabecular number and separation.

### Statistical and power analysis

All values in the animal studies were normalised to the vehicle control. Results are expressed as percentage difference of the mean value from vehicle control. Statistical differences were assessed using the Student's *t* test for non-paired samples in the calvarial *in vitro* studies. In the *in vivo* animal studies, the paired Student's *t* test was used to compare data between the experimental groups. For comparisons between more than two groups of data, such as different treatment concentrations, one-way ANOVA was used followed by Dunnett's test. For all analyses, differences were considered significant at *P* < 0·05.

## Results

### Anti-resorptive screening experiments

Anti-resorptive formula development was initiated by determining the inhibitory effect of botanical extracts on RANKL expression in osteoblast-like human MG-63 cells stimulated with IL-1β. The inhibitory effects of the botanical extracts on RANKL expression are summarised in Table 4. The most potent inhibitory extracts were gingko (31 %), rehmannia (74 %), eleuthero (50 %) and sophora (42 %).

**Table 4.** Effect of botanical extracts (1 µg/ml) on inhibition of IL-1β (10 ng/ml)-stimulated receptor activator of nuclear factor-κB ligand (RANKL) expression in MG-63 cells

Botanicals	RANKL (% change)*
Dong quai	↓ 16
Eleuthero	↓ 50
Ginkgo	↓ 31
Grape seed	↓ 11
Green tea	↓ 19
Pomegranate	↓ 14
Rehmannia	↓ 74
Sophora	↓ 42
Ipriflavone (control)	No effect

↓, Decrease.

* Data are expressed as percentage change in RANKL expression compared with IL-1β-treated controls.

**Fig. 1.**
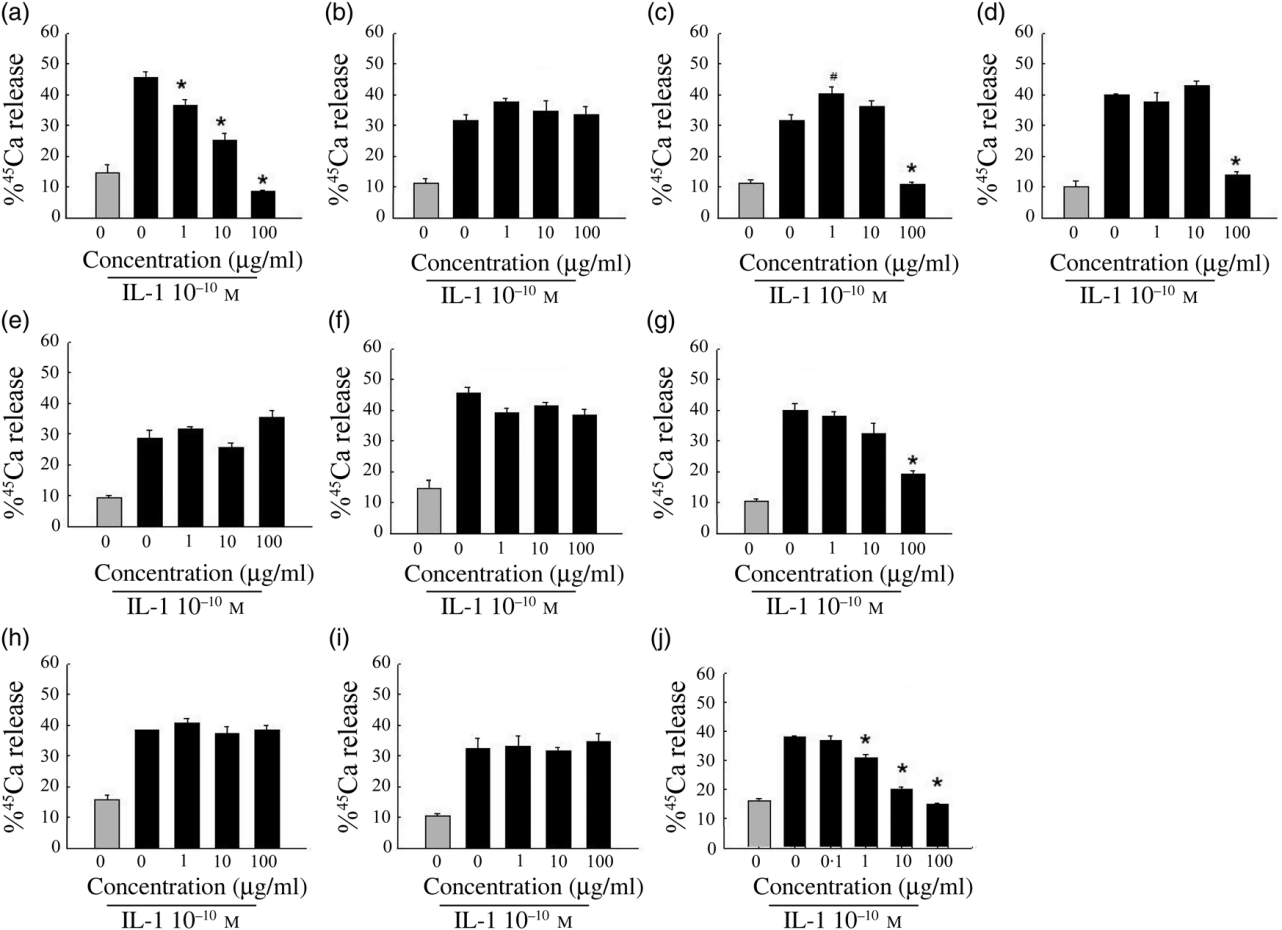
Effect of botanical extracts on calcium release from murine calvarial tissues harvested and incubated with botanical extracts and IL-1 β (10 ng/ml) for 3 d. (a) Pomegranate; (b) ginkgo; (c) green tea; (d) grape seed; (e) rehmannia; (f) eleuthero; (g) ipriflavone; (h) dong quai; (i) sophora; (j) alendronate (positive control). Results are expressed as percentage of ^45^Ca release compared with total amount of calcium present in calvariae. (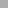), Calcium release without IL-1β stimulation; (■), calcium release with IL-1β stimulation. Values are means, with standard errors represented by vertical bars. * Significant reduction in calcium release compared with IL-1β treatment alone (*P* < 0·05).

The botanical extracts that showed the greatest inhibition of RANKL gene expression were further tested for their ability to inhibit bone resorption in the murine calvarial assay. In this assay, IL-1β stimulated a loss of ^45^Ca from calvarial tissue between 10 to 40 % of baseline. As shown in Fig. 1, the most effective extract in reducing the IL-1β-stimulated Ca release was pomegranate fruit extract; it demonstrates a concentration-dependent inhibition of ^45^Ca release. Extracts of green tea and grape seed, and ipriflavone, a synthetic isoflavone, caused significant inhibition but only at the highest concentration tested (100 µg/ml). Extracts of rehmannia, ginkgo, sophora, dong quai and eleuthero were not effective at any concentration tested. Alendronate, a positive control, also inhibited the IL-1β-stimulated Ca release in a concentration-dependent manner.

**Fig. 2.**
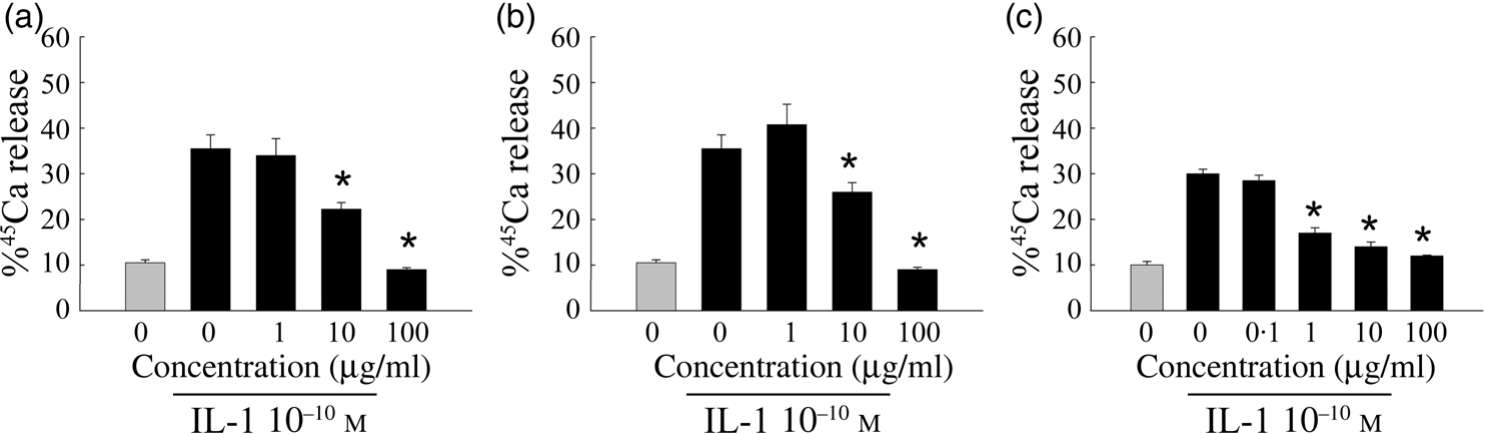
Effect of combinations of pomegranate fruit (P) and grape seed (GS) extracts on calcium release from murine calvarial tissues harvested and incubated with extract combinations and IL-1β (10 ng/ml) for 3 d. (a) P–GS combination (10:1); (b) P–GS–ipriflavone combination (43:4·3:52); (c) alendronate positive control. Results are expressed as percentage of ^45^Ca release compared with total amount of calcium present in calvariae. (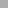), Calcium release without IL-1β stimulation; (■), calcium release with IL-1β stimulation. Values are means, with standard errors represented by vertical bars. * Significant reduction in calcium release compared with IL-1β treatment alone (*P* < 0·05).

Additional calvarial studies were performed to identify optimal combinations of the best-performing botanical extracts. A combination of pomegranate fruit and grape seed extracts (10:1) showed a concentration-dependent inhibition of IL-1β-stimulated Ca loss (Fig. 2). A combination of pomegranate fruit extract, grape seed extract and ipriflavone (43:4·3:52) also demonstrated a concentration-dependent inhibition of Ca loss (Fig. 2).

The pomegranate fruit–grape seed–ipriflavone combinations tested in the calvarial anti-resorption and bone formation assays were also tested *in vivo* (Table 2). The combinations of pomegranate fruit and grape seed extracts were administered to rats in doses of 56, 140 and 223 mg/kg body weight (as listed in Table 2 for anti-resorptive (AR) samples AR-1 to AR-3). Ipriflavone was administered at a dose of 61 mg/kg and in combination with pomegranate and grape seed in total amounts of 117 and 201 mg/kg body weight (samples AR-4 to AR-6).

Bone histomorphology was performed using sections of the proximal tibia examined for the percentage of bone area compared with total tissue and as percentage change from vehicle. As expected, cancellous bone mass was significantly reduced (127·9 %) in femurs of vehicle-treated OVX rats relative to vehicle-treated SHAM controls. Alendronate prevented bone loss associated with OVX and caused an increase in bone area compared with the vehicle-treated control. The only mixture of botanical extracts to cause a significant increase in bone area (percentage bone area/total volume) was the combination of pomegranate fruit and grape seed extracts (sample AR-1), with an increase of 61·4 % when compared with the control group (Table 2).

In order to determine strength, stiffness and resistance to fracture of excised long bones, a three-point bending biomechanical test was performed. Two combinations of pomegranate and grape seed extracts, test samples AR-2 and AR-3, showed a significant increase in the maximum force required to fracture bone compared with the vehicle control, while the combination of pomegranate fruit and grape seed extracts (AR-3) was the only mixture to significantly increase the stiffness of the femur compared with control (Table 2). The other treatments showed no significant difference in these parameters in OVX animals compared with vehicle-treated controls. In addition, alendronate, the positive control, had no effect on stiffness or the ability of the bones to withstand fracture. All but the lowest concentration mixture of pomegranate fruit and grape seed extracts including alendronate significantly increased BMD as compared with the vehicle-treated controls.

For the µCT40 measurements, alendronate significantly increased vBMD, percentage bone volume/total volume and trabecular number, and decreased trabecular separation, as expected. Among the experimental treatments, the pomegranate fruit–grape seed combinations (AR-1 to AR-3) showed similar effects as alendronate in all µCT measurements but at a lower magnitude (Table 2). In addition to the significant effects on vBMD, percentage bone volume/total volume, and trabecular number and separation, an apparent dose–response was observed for the pomegranate fruit–grape seed combinations.

### Bone formation screening experiments

A total of five botanical extracts were evaluated for their effects on BMP-2 promoter activation, and increased BMP-2 mRNA and protein expression (Table 5). All five extracts increased BMP-2 gene expression from 3- to 49-fold over untreated control. Extracts of fava d'anta (quercetin) and sophora caused significant increases in BMP-2 luciferase promoter activity. A significant increase in BMP-2 protein levels was seen following treatment with eleuthero, quercetin and sophora extracts.

**Table 5.** Effect of botanical extracts on bone morphogenetic protein-2 (BMP-2) luciferase promoter activity, and mRNA and protein expression in MG-63 cells

		Sample concentrations achieving significant effect (µg/ml)	
Sample	BMP-2 mRNA* (fold increase)	BMP-2 promoter†	BMP-2 protein synthesis‡
Eleuthero	27	–	1, 10, 100
Licorice	45	–	–
Quercetin	3	6·3, 12·5, 25, 50, 100	1, 10, 100
Rehmannia	28	–	–
Sophora	49	100	1, 10, 100
Ipriflavone	–	3·2, 6·3, 12·5, 25, 50, 100	1
Orthosilicic acid	Not tested	0·5 µm	Not tested
Simvastatin	Not tested	5 µm	Not tested

* BMP-2 expression levels were measured for MG-63 cells treated with sample extracts at 10 µg/ml. Data are expressed as fold change in BMP-2 mRNA compared with controls. A greater than two-fold increase is considered significant.

† BMP-2 promoter assay was measured using a clonal osteoblast cell line (2T3 cells) that was stably transfected with murine BMP-2 promoter (–2712/ +165) linked to firefly luciferase cDNA. The sample concentrations listed significantly increased (*P* < 0·05) luciferase activity compared with controls.

‡ BMP-2 protein levels were measured from supernatant fractions of treated MG-63 cells using an ELISA assay. The sample concentrations listed significantly increased (*P* < 0·05) protein synthesis compared with controls.

**Fig. 3.**
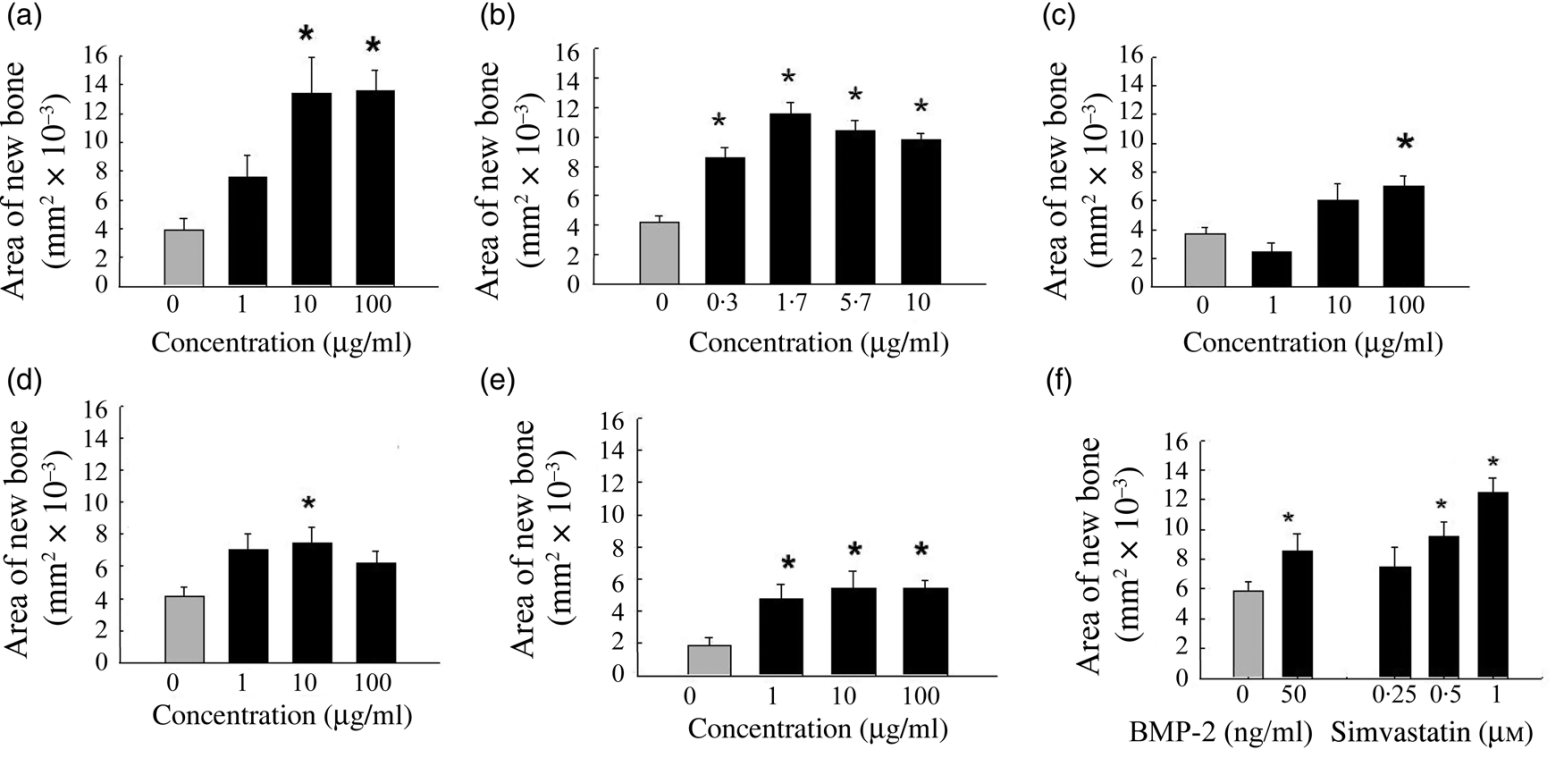
Effect of botanical extracts on bone formation in murine calvarial tissues. Calvarial tissues were harvested, incubated with botanical extracts for 7 d, and examined using histomorphometric analysis. (a) Quercetin; (b) licorice; (c) ipriflavone; (d) sophora; (e) eleuthero; (f) positive controls (bone morphogenetic protein-2 (BMP-2) and simvastatin). Results are expressed as percentage of area of new bone (mm^2^ × 10^−3^). (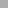), Control (untreated) samples. Values are means, with standard errors represented by vertical bars. * Significant increase in area of new bone formation as compared with controls (*P* < 0·05).

On the basis of the results from the bone formation screen, quercetin, licorice, sophora and eleuthero extracts were selected for further testing in the calvarial bone formation assay. Simvastatin, licorice extract, quercetin and eleuthero extracts were shown to stimulate bone growth in a concentration-dependent manner (Fig. 3).

**Fig. 4.**
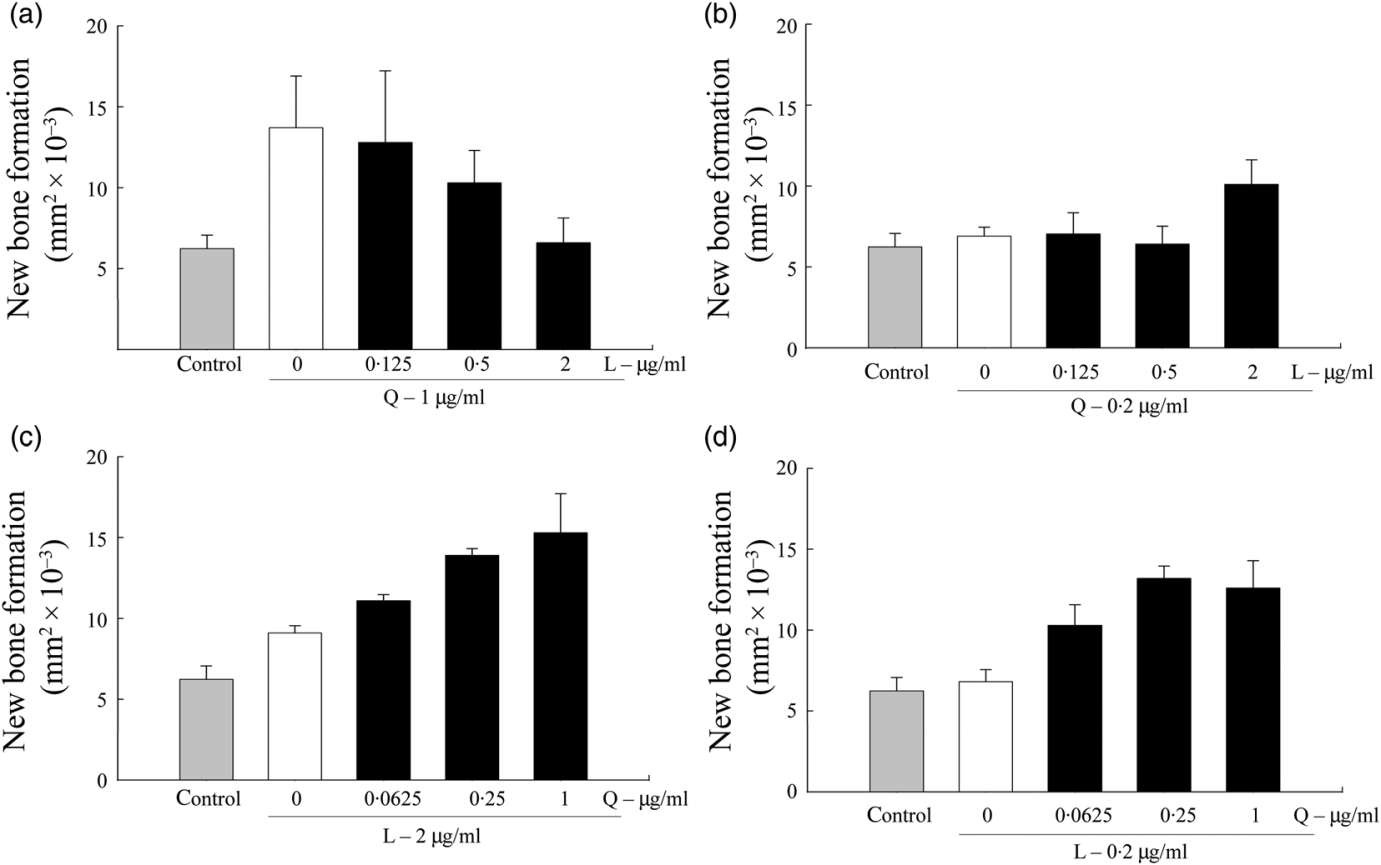
Effect of combinations of quercetin (Q) and licorice (L) extract on bone formation in murine calvarial tissues. Calvarial tissues were harvested, incubated with botanical extracts for 7 d, and examined using histomorphometric analysis. The concentration of either quercetin or licorice extract was kept constant while the other extract concentration was varied. (a) Quercetin constant at 1 µg/ml; (b) quercetin constant at 0·2 µg/ml; (c) licorice constant at 2 µg/ml; (d) licorice constant at 0·2 µg/ml. Results are expressed as percentage of area of new bone (mm^2^ × 10^−3^). (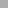), Control (untreated) samples. Values are means, with standard errors represented by vertical bars.

To identify optimal combinations of botanical extracts on bone formation, we tested combinations of sophora, eleuthero and licorice extracts, quercetin and ipriflavone in the cultured murine calvarial assays. The results from the various combinations of extracts suggested that a combination of licorice extract and quercetin was the most effective at increasing new bone formation in the calvarial assay (Fig. 4).

To further determine the efficacy of combinations of quercetin and licorice extracts on bone health, we analysed their effects in intact Sprague–Dawley female rats using histomorphology, and biomechanical and μCT measurements.

Histomorphometric analysis in the proximal tibiae demonstrated a significant increase in bone volume as well as bone formation rate in PTH-treated control rats. Of the six botanical combinations tested, bone formation (BF) sample BF-3 (25 mg/kg body weight quercetin and 13 mg/kg body weight licorice extract) showed the most significant changes, with effects similar to those of the PTH-positive control (Table 3).

There were no increases in the biomechanical measurements (stiffness and maximum force) for any samples tested, including PTH. Treatment with PTH has been shown to increase biomechanical measurements of bone strength in studies of longer duration (60 to 180 d)^(^^18^^)^. Our lack of response in bone formation may be due to the relatively short duration of treatment (35 d).

The µCT analysis showed an 86 % increase in vBMD by PTH as compared with vehicle control. Quercetin and licorice extract combinations BF-2 and BF-5 showed significant differences in all bone measurements as compared with vehicle control.

### Animal health

None of the samples tested caused a change in average weight gain or a change in food consumption over the course of the experiments.

## Discussion

Our strategy for developing anti-resorptive and bone formation formulas was based on molecular targets for these opposing mechanisms. This research suggests that combinations of pomegranate fruit and grape seed extracts may mitigate bone resorption while combinations of licorice extract and quercetin may enhance bone formation.

Research on the therapeutic properties of pomegranate extracts has focused on its antioxidant, anti-inflammatory and anti-cancer potential^(^^19^^–^^21^^)^. Recent research also supports a role for pomegranate extracts on bone health^(^^22^^–^^24^^)^. A study of the effects of pomegranate extract on bone health in OVX mice showed no significant decrease in BMD in the distal femur and proximal tibia as compared with the intact mice, while control OVX mice had a significant loss of BMD. Of the other bone parameters studied, only trabecular separation was significantly increased in the OVX mice; however, the OVX mice treated with pomegranate extract had trabecular separation similar to that of intact mice^(^^22^^)^. These results support our findings, although the present results were for mixtures of pomegranate fruit and grape seed extracts rather than pomegranate extract alone.

A study using arthritis-induced mice demonstrated that pomegranate extract containing 86 % ellagitannins resulted in statistically significant reductions in synovitis, pannus and bone erosion scores when compared with water-fed mice. Histopathological analysis indicated a reduction in joint infiltration by inflammatory cells^(^^23^^)^. Pregnant mice fed pomegranate fruit or husk extracts between days 8 and 18 of gestation showed increased Ca content in the pregnant mice as well as increased femur length and indices of osteogenesis in the embryos^(^^24^^)^. Purified ellagic acid has also been shown to stimulate mineralisation of extracellular matrix by the osteoblastic cell line, KS483 cells, similar to that of 17β-oestradiol^(^^25^^)^. These studies are in agreement with our findings that pomegranate fruit extract, standardised to 40 % ellagic acid, inhibits IL-1β-induced RANKL expression in MG-63 human osteosarcoma cells.

Grape seed extracts are used in dietary supplements for their antioxidant activity. Two rat studies have explored the bone health benefits of grape seed proanthocyanidins^(^^26^^,^^27^^)^. A grape seed extract and Ca-supplemented diet was shown to increase bone density, mineral content and bone strength in Ca-deficient rats at a greater level than Ca supplementation alone^(^^26^^)^. Grape seed extract in combination with Ca has also been shown to be effective in reversing mandibular condyle bone debility in rats beyond that of Ca supplementation alone^(^^27^^)^. Grape seed extract has also been shown to inhibit osteoclastogenesis in bone marrow cells cultured with RANKL and macrophage colony-stimulating factor^(^^28^^)^. In the present study, we demonstrated that grape seed extract inhibits IL-1β-induced RANKL expression in MG-63 human osteosarcoma cells and significantly reduces Ca loss from mouse calvariae.

In combination, pomegranate fruit and grape seed extracts prevented OVX-induced bone loss in rats. This effect appears to be dose-dependent, with a 10:1 combination of pomegranate and grape seed being the most effective at preventing bone loss (Table 2 and Fig. 2).

In the last decade, there have been more than sixty clinical studies on the efficacy of ipriflavone for the prevention and reversal of bone loss. It has been shown that a dose of 600 mg/d improves bone density and prevents bone loss in premenopausal women^(^^29^^)^. In the present study, we found that an equivalent dose of ipriflavone increased BMD, but not bone strength, when administered to OVX rats. When ipriflavone was added to a combination of pomegranate fruit and grape seed extracts, it did not increase their effectiveness on any of the bone measurements (Table 2). In the present research, we found that a 10:1 combination of pomegranate fruit and grape seed extracts was the most effective anti-resorptive formula with a mechanism of action, at least in part, through the inhibition of RANKL.

The most efficacious bone formation formula was a combination of quercetin and licorice extracts. Quercetin, commonly found in food plants such as onions, apples and blueberries, has been shown to have antioxidant, anti-inflammatory, antiviral, immunomodulatory, anti-cancer and gastroprotective properties^(^^2^^)^. Quercetin given as 0·25 % of the diet has been shown to preserve bone density in OVX female mice^(^^30^^)^. It has also been shown to suppress osteoclastogenesis and RANKL-induced NF-κB activation in osteoclast precursors. In contrast, the research also showed that quercetin antagonised both transforming growth factor-β and BMP-2 induced Smad activation in osteoblast precursors, suggesting that quercetin might mediate stimulatory and inhibitory actions on osteoblasts resulting in a balance between the two functions^(^^31^^)^. Another study suggested that quercetin stimulates osteoblastic activity measured as an increase in alkaline phosphatase activity in MG-63 human osteoblasts^(^^32^^)^. In the present research, we demonstrated that quercetin increases BMP-2 promoter activity, and BMP-2 mRNA and protein expression (Table 5). Quercetin was also found to significantly increase new bone growth in a murine calvarial assay (Fig. 3).

It has been proposed that quercetin may have oestrogen-like effects due to its ability to bind the oestrogen receptor^(^^33^^,^^34^^)^ and, thus, may have the potential to counteract the deleterious effects of oestrogen deficiency on bone. Glabridin from licorice root has also been reported to have oestrogenic activity^(^^35^^,^^36^^)^. A role for glabridin in bone health was indicated in a study in which it stimulated creatine kinase-specific activity in diaphyseal bone and epiphyseal cartilage in prepubertal female rats. In OVX female rats, a single injection of glabridin (100 µg) increased creatine kinase-specific activity similar to the increase seen with administration of 17β-oestradiol^(^^36^^)^. Glabridin has also been shown to increase cell growth, alkaline phosphatase activity, collagen content and osteocalcin secretion in a mouse osteoblastic cell line, MC3T3-E1^(^^35^^)^. Orthosilicic acid, soluble silica, has been found to stimulate collagen type I synthesis and osteoblastic differentiation in human osteoblast-like cells *in vitro*^(^^37^^)^.

In our bone formation assays, quercetin extract stimulated the BMP-2 promoter, and BMP-2 mRNA and protein expression *in vitro* (Table 5), which is indicative of increased bone formation. The increased bone formation is further indicated in the murine calvarial assays where the combination of quercetin and licorice extract promoted an increase in new bone area. When the amount of licorice extract was held constant, there was a concentration-dependent increase in new bone formation with increasing amounts of quercetin; however, when the amount of quercetin was held constant the licorice extract did not show a similar concentration-dependent response (Fig. 4).

The results of the *in vivo* assays did not point to a single most effective combination of quercetin and licorice extract. The histomorphology results were significant for the 25·4 mg quercetin and 12·7 mg licorice combination (BF-3). The µCT measurements resulted in significant effects for the BF-2 (50·8 mg of each ingredient) and BF-5 (101·7 mg quercetin and 50·8 mg licorice) formulations. The lack of an apparent dose–response relationship with BF-3, BF-4 and BF-5 formulations is puzzling. These results are intriguing, and illustrate both the complexity of bone formation and the need for further research to elucidate the efficacy of quercetin and licorice extracts on these bone measurements.

In this systematic approach for the selection of botanical extracts that support bone health, we describe a targeted series of *in vitro* and *in vivo* assays that result in two different botanical extract formulas that affect bone resorption and formation. The anti-resorptive formula of pomegranate fruit and grapeseed extracts is designed to maintain bone mass by preventing Ca loss, where the bone formation formula of quercetin and licorice extracts assists in the maintenance of maximum bone mass by stimulating bone formation and enhancing Ca deposition. This series of assays represents the initial steps in determining the potential of natural plant extracts as therapeutic modalities to improve bone health in patients. Clearly, these results are limited in terms of directly extrapolating the results from rodents to humans. However, given the nature of these extracts and the limited understanding of their efficacious pathways, it is intriguing to see these extract combinations eliciting bone-protective and bone-building effects. Future clinical studies are needed to confirm these effects in human subjects.
